# Differential fate and functional outcome of lithium chloride primed adult neural progenitor cell transplants in a rat model of Huntington disease

**DOI:** 10.1186/scrt41

**Published:** 2010-12-22

**Authors:** Elena M Vazey, Bronwen Connor

**Affiliations:** 1 Department of Pharmacology and Clinical Pharmacology, Centre for Brain Research, Faculty of Medical Health Sciences, The University of Auckland, Private Bag 92019, Auckland Mail Centre, Auckland 1142, New Zealand; 2 Current address: Department of Neurosciences, Medical University of South Carolina, BSB 403, 173 Ashley Avenue, Charleston, SC, 29403, USA

## Abstract

**Introduction:**

The ability to predetermine the fate of transplanted neural progenitor cells (NPCs) and specifically to direct their maturation has the potential to enhance the efficiency of cell-transplantation therapy for neurodegenerative disease. We previously demonstrated that transient exposure of subventricular zone (SVZ)-derived adult NPCs to lithium chloride during *in vitro *proliferation alters differential fate *in vitro *and increases the proportion of cells expressing neuronal markers while reducing glial progeny. To extend these findings, we examined whether *in vitro *priming of adult SVZ-derived NPCs with lithium chloride before transplantation into the quinolinic acid (QA) lesion rat model of Huntington disease altered *in vivo *neuronal differentiation and sensorimotor function compared with nonprimed NPC transplants.

**Methods:**

NPCs were isolated from the SVZ of the adult rat brain and cultured for 2 weeks. Four days before transplantation into the QA-lesioned rat striatum, the cells were labeled with BrdU and primed with lithium chloride. The rats underwent regular evaluation of forelimb use and sensorimotor neglect to establish functional effects of NPC transplantation. Twelve weeks after transplantation, the brains were analyzed with immunohistochemistry to compare the differential fate of primed and nonprimed NPCs.

**Results:**

We observed that *in vitro *priming of adult NPCs with lithium chloride reduced gliogenesis and enhanced the occurrence of DARPP-32-positive neurons when compared with nonprimed cells 12 weeks after transplantation into the QA-lesioned striatum. Lithium chloride priming also augmented the formation of efferent projections from newly formed neurons in the damaged host striatum to the globus pallidus. This was associated with acceleration of sensorimotor function recovery in rats receiving transplants of lithium chloride-primed adult NPCs compared with nonprimed transplants.

**Conclusions:**

These initial findings indicate that *in vitro *priming of adult NPCs with lithium chloride may augment transplant efficiency and accelerate sensorimotor function outcome *in vivo*.

## Introduction

Cell-transplantation therapy offers a viable treatment strategy for a range of neurodegenerative disorders, including Huntington disease (HD) [1-3]. Cell transplantation for HD has developed over the last decade to clinical trials of human fetal striatal tissue in the United States, France, and the United Kingdom [4]. However, the supply of human fetal tissue is limited and difficult to develop as a routine systematized source. This has led to neural progenitor cell (NPC) populations being investigated as an alternative source of cells for transplantation therapy for HD. Although initial studies have investigated NPCs obtained from a range of sources, they have all demonstrated limited mature neuronal differentiation with varying levels of sensorimotor function improvement [5-9]. Specifically, we previously demonstrated that subventricular zone (SVZ)-derived adult NPCs survive transplantation, generate new striatal neurons, and improve sensorimotor function in the quinolinic acid (QA)-lesion model of HD [9]. However, in agreement with other studies [10-14], we observed the majority of transplanted adult NPCs generated glial fibrillary acidic protein (GFAP)-positive astrocytes. Regional environmental cues present in the adult brain play a key role in determining the lineage potential of NPCs [10,12,13,15]. We propose that microenvironmental changes induced by a CNS lesion in non-neurogenic brain regions are not sufficient to induce extensive neuronal differentiation from transplanted NPCs. As limited neuronal differentiation of transplanted NPCs may result in incomplete functional recovery, it is necessary to develop an effective strategy by which the neuronal fate of NPCs can be predetermined before transplantation.

We recently established an effective strategy that allows us to "prime" adult NPCs toward a neuronal cell fate in a controlled *in vitro *environment before transplantation. We demonstrated that priming SVZ-derived adult NPCs with lithium chloride increased the proportion of cells expressing neuronal markers while reducing glial progeny [16]. The use of lithium chloride as a priming agent to promote neuronal differentiation is supported by previous studies that demonstrate the ability for lithium chloride to increase neuronal differentiation of both fetal and adult hippocampal NPC cultures *in vitro*, as well as to augment adult hippocampal neurogenesis *in vivo *[17-19].

The current study extends our previous findings by examining whether *in vitro *priming of adult SVZ-derived NPCs with lithium chloride before transplantation into the QA lesion rat model of HD can enhance *in vivo *neuronal differentiation and sensorimotor function recovery.

## Materials and methods

### Animals

Adult male Wistar rats (University of Auckland Vernon Jansen Unit) 250 to 300 g (8 weeks old) at the time of QA lesion were used in this study. All procedures strictly complied with the University of Auckland Animal Ethics Guidelines, in accordance with the New Zealand Animal Welfare Act 1999 and international ethical guidelines. All efforts were made to minimize the number of animals used and their suffering. The rats were randomly allocated into the following treatment groups: nonprimed NPC transplants (*n *= 12); lithium chloride-primed NPC transplants (*n *= 11); and sham transplants (*n *= 8). The animals were group housed in a temperature- and humidity-controlled room on a 12-hour light/dark cycle. Food and water were available *ad libitum *throughout the study, except for food-deprivation requirements for motor-function analysis, as noted later.

### Isolation, expansion, and priming of adult neural progenitor cells

Neural progenitor cells (NPCs) were isolated from the SVZ of adult male Wistar rats, 250 to 300 g, and cultured as previously described [9,11,16] with retinoic acid-free B27 (Invitrogen, Carlsbad, CA, USA). To visualize transplanted adult rat NPCs and their progeny in the host rat brain, neurospheres were labeled with bromodeoxyuridine (BrdU; 5 μ*M*; Applichem, Darmstadt, Germany) from days 10 to 14 of culture [9,11,16]. We previously confirmed that BrdU labeling of transplanted NPCs does not result in transfer of BrdU from dying NPCs to host cells [11]. Priming was performed by adding lithium chloride (3 m*M *final concentration) to the proliferation medium from days 10 to 14 of culture [16]. We previously confirmed that treatment with lithium chloride has no effect on NPC proliferation or viability when compared with nonprimed NPC cultures [16]. The NPCs were harvested mechanically at day 14, rinsed twice to remove residual BrdU, and resuspended for transplantation in L15 (Invitrogen) with 6 mg/ml *d*-glucose at ~37,500 cells/μl [9,11].

### Surgical procedures

All surgeries were performed after an intraperitoneal (i.p.) injection of sodium pentobarbital (60 mg/kg). All rats received a unilateral intrastriatal infusion of quinolinic acid (QA; 50 nmol, 400 nl; 100 nl/min with a 32G Hamilton syringe controlled by a WPI UltraMicroPump II) at the following stereotaxic coordinates: +0.7 mm anterior-posterior (AP), ± 2.7 mm medial-lateral (ML), relative to bregma, and -5.0 mm dorsal-ventral (DV) from dura. Injections of NPCs (~150,000 viable cells per animal) or L15 alone (sham) were performed 21 days after QA lesioning into two adjacent sites in the lesioned striatum (~37,500 viable cells/μl; 2 μl per injection site; 400 nl/min with a 26G Hamilton syringe) at the following stereotaxic coordinates: +0.5 mm AP, ± 2.5 mm ML, at -5.0 and -4.0 mm DV [9,11]. To assess whether transplanted adult NPCs formed anatomically appropriate efferent projections, the neuronal tracer hydroxystilbamidine, Fluorogold (0.2 μl of 2% solution; FG; Invitrogen) was injected into the ispilateral globus pallidus 11 weeks after transplant at the following stereotaxic coordinates: -0.9 mm AP, ±3.5 mm ML, and -6.0 mm DV.

### Sensorimotor function analysis

Functional sensorimotor performance in the spontaneous exploratory forelimb-use test [20] and corridor task [21-25] was assessed 1 week before QA lesioning, 3 weeks after QA lesioning (before transplantation), and periodically for 12 weeks after transplantation by an investigator blinded to treatment. Forelimb use was assessed as a single asymmetry score representing the overall ipsilateral forepaw use for rearing, wall placement, and landing during exploratory rearing over a 5-minute trial period in a clear Perspex cylinder (200 mm internal diameter). The corridor task assessed ipsilateral retrieval bias/contralateral sensorimotor neglect with left/right food selection from adjacent containers evenly spaced along a narrow corridor with retrievals analyzed as ipsilateral or contralateral to the treated striatum. The first 20 retrieval attempts (5 minutes maximum) were recorded on each testing occasion. Rats were subjected to fasting by food withdrawal for 12 hours before corridor testing to elicit enhanced appetite for motivation.

### Immunohistochemical analysis

Rats were killed 12 weeks after transplantation with sodium pentobarbital (120 mg/kg i.p.) followed by transcardial perfusion with 0.9% saline and 4% paraformaldehyde. Brains were cryoprotected in 30% sucrose before sectioning at 40 μm on a HM450 sliding microtome (Microm International GmbH, Walldorf, Germany). Eight sets of sections were collected from each brain (distance of 320 μm between consecutive sections in each set) and stored at -20°C.

Diaminobenzidine (DAB) peroxidase immunohistochemistry was performed on free-floating coronal sections from each animal to identify transplanted BrdU-labeled NPCs (mouse anti-BrdU, 1:250; Billerica, MA, USA). DNA was denatured in 2N hydrochloric acid at 37°C for 60 minutes and rehydrated in 0.1 *M *sodium tetraborate (pH 8.5). The sections were blocked in Tris-buffered saline containing 0.1% Triton X-100 with 3% normal goat serum. After incubation with the primary antibody, a biotinylated secondary antibody (1:500; goat anti-mouse; Sigma-Aldrich, St. Louis, MO, USA) was added before incubation in ExtrAvidin peroxidase (1:500; Sigma-Aldrich). Antibodies were visualized by using 0.4 mg/ml DAB, 25 mg/ml nickel sulfate, and 0.005% hydrogen peroxide in 0.2 *M *phosphate buffer.

Primary antibody dilutions for double-label immunofluorescence were 1:250 for BrdU (mouse anti-BrdU Alexa Fluor 594; Invitrogen), 1:250 for DARPP-32 (rabbit anti-DARPP-32; Millipore), 1:500 for GFAP (rabbit anti-GFAP; DAKO), 1:500 for NeuN (mouse anti-NeuN; Millipore), and 1:50 for SOX2 (rabbit anti-SOX2; R & D Systems, Minneapolis, MN, USA). In the detection of BrdU labeling, free-floating sections first underwent DNA denaturation, as described earlier, before serial incubation with appropriate primary antibodies. Secondary goat anti-rabbit and goat anti-mouse Alexa Fluor 488 conjugated antibodies (1:500; Invitrogen) were used. Fluorescently labeled sections were imaged by using a confocal laser-scanning microscope (Leica TCS SP2) equipped with UV, argon, argon/krypton, and helium/neon lasers (Biomedical Imaging Resource Unit, University of Auckland). All figures were compiled by using Adobe Photoshop (Adobe Systems, Inc., San Jose, CA, USA).

### Quantification

The number and distribution of BrdU-labeled adult NPCs present in the QA-lesioned adult rat brain 12 weeks after transplantation were measured on DAB/nickel sulfate-stained sections labeled for BrdU. To determine the distribution of transplanted NPCs in the lesioned hemisphere, an entire series from every animal (320 μm distance between each section) was systematically analyzed at 20× magnification from both the anterior (bregma +4.5 mm) and posterior maxima (bregma -8.0 mm) inward until the first BrdU-labeled cell nucleus and process was identified and recorded in stereotaxic coordinates.

The number of BrdU-labeled NPCs in the QA-lesioned hemisphere 12 weeks after transplantation was quantified by using MicroBrightfield Neurolucida software (Williston, VT, USA) on a Nikon e800 microscope with MicroFire S99808 digital camera (Optronics, Muskogee, OK, USA). Virtual slices were created at 20× magnification of the ipsilateral hemisphere in one series (bregma +2.20 mm to bregma -0.92 mm) from each animal that received an NPC transplant. With NIH Image J, the virtual slices underwent semiautomated analysis based on intensity thresholding and particle size to determine the number of BrdU-positive particles. Counts from one section per animal were verified with a manual count by using Neurolucida software. Automated counts were then extrapolated by using Abercrombie's correction [26].

Fluorescent double-label analysis was undertaken on a Leica TCS SP2 confocal laser scanning microscope (Biomedical Imaging Resource Unit, University of Auckland). Colocalization of BrdU and a cell marker or Fluorogold was confirmed with z-series (interslice gap, 0.28 μm) and orthogonal reconstruction through the cell of interest. For quantification, a total of 500 transplanted cells per animal within anterior, medial, and posterior sections of the lesioned striatum for each phenotypic marker were manually identified with confocal microscopy. For FG analysis, all cells counted were from anterior and medial sections of the striatum at least 1 mm rostral of the injection site to avoid confound due to passive diffusion. Each channel was imaged sequentially with collection from selective emission spectra to reduce cross-excitation or bleed-through of fluorophores. Analysis was undertaken blind to the treatment of each animal.

### Statistical analysis

**Figure 1:**
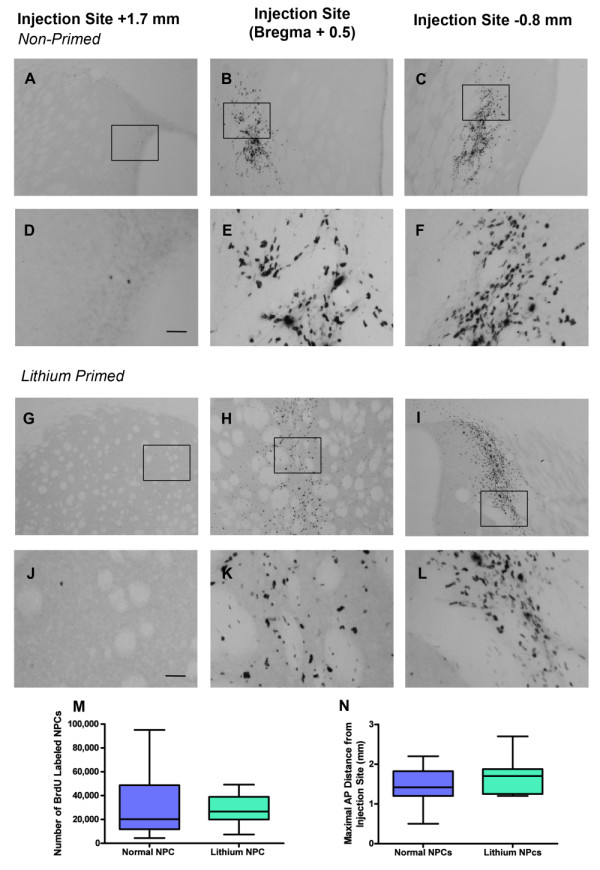
**BrdU-labeled cells were found throughout the rostrocaudal axis of the QA-lesioned striatum 12 weeks after transplantation of nonprimed (a through f) and lithium chloride-primed (g through l) transplants**. No difference was found in the number **(m) **or distribution **(n) **of BrdU-labeled cells between lithium chloride-primed and nonprimed transplants (Tukey box plot with min/max inner fences). Boxed regions in (a through c) and (g through i) indicate the regions imaged in (d through f) and (j through l), respectively. Scale bar: (a through c) and (g through i), 100 μm; (d through f) and (j through l), 25 μm. **P < 0.01.

Statistical analysis and graph composition was undertaken by using GraphPad Prism version 4.02 for Windows (GraphPad Software, San Diego, CA, USA). In text, values and bar graphs are presented as mean ± standard error of the mean (SEM); some graphs (Figure 1m and 1n) are presented as Tukey box-and-whisker plots. Two-tailed unpaired Student *t *tests were used for direct comparisons between primed and nonprimed NPCs. One-way or two-way ANOVAs were used for multiple group comparisons with Tukey or Bonferroni *post hoc *tests, respectively. Statistical significance was established at *P *< 0.05.

## Results

### Survival and migration of transplanted adult neural progenitor cells

Neural progenitor cells (NPCs) were isolated from the SVZ of the adult rat brain and cultured in proliferative conditions for 14 days, including 4 days of priming with lithium chloride. As we previously demonstrated [16], lithium chloride priming had no effect on NPC proliferation or viability when compared with nonprimed NPC cultures. To enable detection of adult NPCs after transplantation into the QA-lesioned rat striatum, cells were labeled with BrdU 4 days before harvest. With CldU and IdU co-labeling, we identified that the majority of SVZ-derived adult NPCs exhibit a cell cycle of 18 to 26 hours. In agreement with previous studies [9,11], this results in labeling of 83.86 ± 10.18% of NPCs with BrdU, with no difference in labeling efficiency observed between primed and nonprimed cultures (*P *= 0.79).

Twelve weeks after transplantation, BrdU-labeled NPCs were distributed across the rostrocaudal axis of the QA-lesioned striatum in all transplanted animals (Figure 1a through 1l). Quantification of BrdU-labeled cell number in the ipsilateral hemisphere between AP +2.20 mm and -0.92 mm (relative to bregma) determined that lithium chloride priming did not augment the survival of transplanted adult NPCs (32,680 ± 9,663 nonprimed (27%) versus 38,216 ± 13,113 primed (32%); *t*_20 _= 0.33; *P *= 0.74; Figure 1m). Furthermore, lithium chloride priming did not alter the maximal distribution of NPCs across the striatum (AP +4.5 mm to -8.0 mm relative to bregma) compared with nonprimed cells (nonprimed = 1.5 ± 0.09 mm; primed = 1.67 ± 0.09 mm; *t*_41 _= 1.32; *P *= 0.19; Figure 1n).

### Neuronal differentiation of transplanted adult neural progenitor cells

**Figure 2:**
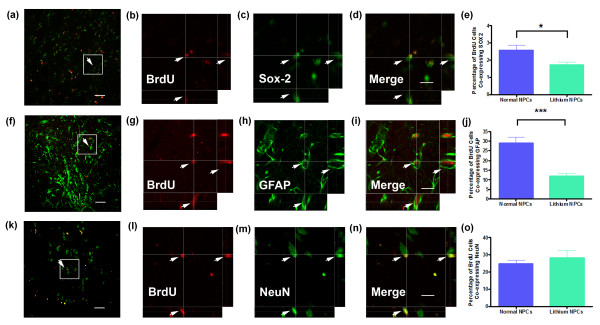
***In vitro *priming of SVZ-derived adult neural progenitor cells with lithium chloride reduces gliogenesis 12 weeks after transplantation**. Representative confocal micrographs in low power and in high power with orthogonal projections of BrdU-labeled lithium chloride-primed adult neural progenitor cells co-expressing **(a **through **d) **the immature neural progenitor cell marker SOX-2, **(f **through **i) **the astrocytic marker GFAP, and **(k **through **n) **the mature neuronal marker NeuN. Graphs comparing the proportion of transplanted BrdU-labeled cells co-expressing **(e) **SOX2, **(j) **GFAP, or **(o) **NeuN labeling 12 weeks after transplantation. Scale bar, 40 μm (a, f, and k) and 20 μm (B-D, G-I, L-N). *P < 0.05; ***P < 0.001.

To determine the resultant phenotype of the adult NPCs 12 weeks after transplantation into the QA-lesioned striatum, we used BrdU labeling and double- or triple-label immunofluorescence for cell type-specific markers. Confocal microscopy revealed that 12 weeks after transplantation, a small population of BrdU-labeled cells in the QA-lesioned striatum co-expressed the immature progenitor cell marker SOX2 (Figure 2a through 2d). BrdU-labeled cells coexpressing SOX2 were observed in both nonprimed and primed transplants. SOX2 is widely expressed by adult rat SVZ-derived NPCs after 14 days of *in vitro *proliferation [9,11,16]. *In vitro *priming with lithium chloride significantly (Welch's corrected *t_22 _*= 2.36; *P *= 0.027) reduced the proportion of transplanted BrdU-labeled cells coexpressing SOX2 (Figure 2e). Rats that received lithium chloride-primed adult NPC transplants exhibited a residual population of 1.82 ± 0.19% BrdU-labeled cells coexpressing SOX2, compared with 2.56 ± 0.3% of BrdU-labeled cells that expressed SOX2 in nonprimed adult NPCs 12 weeks after transplantation. This suggests that *in vitro *priming of adult NPCs with lithium chloride can direct NPCs away from maintaining an immature phenotype *in vivo*.

**Figure 3:**
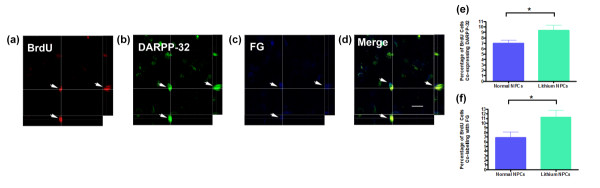
***In vitro *priming of SVZ-derived adult neural progenitor cells with lithium chloride enhances the formation of region-specific mature neurons 12 weeks after transplantation**. **(a **through **d) **Representative confocal micrographs with orthogonal projections of BrdU-labeled lithium chloride-primed adult neural progenitor cells co-expressing DARPP-32, a marker of striatal medium-size spiny projection neurons retrogradely co-labeled with Fluorogold (FG) from the globus pallidus. Graphs comparing the proportion of transplanted BrdU-labeled cells co-expressing **(e) **DARPP-32, or **(f) **FG labeling 12 weeks after transplantation. Scale bar = 20 μm. *P < 0.05.

Throughout the QA-lesioned striatum of rats that received either primed or nonprimed transplants, we observed a population of BrdU-labeled cells exhibiting a mature astrocytic morphology and coexpressing the astroglial marker GFAP (Figure 2f through 2i), with the majority located close to the injection site. In agreement with our previous data [16], *in vitro *priming of adult NPCs with lithium chloride robustly decreased the proportion (Welch's corrected *t*_19 _= 5.16; *P *< 0.0001; Figure 2j) of BrdU-labeled cells coexpressing GFAP from 29.11 ± 3.04% in nonprimed transplants to 11.78 ± 1.42% in primed NPC transplants. An additional population of BrdU-labeled cells within the QA-lesioned striatum expressed the mature neuronal marker NeuN 12 weeks after transplantation of either primed or nonprimed adult NPCs (Figure 2k through 2n). However, *in vitro *priming with lithium chloride did not significantly alter (Welch's corrected *t*_19 _= 0.81; *P *= 0.43) the proportion of BrdU-labeled cells coexpressing NeuN when compared with nonprimed cells (primed, 28.40 ± 4.0%; nonprimed, 24.73 ± 2.2%; Figure 2o). We also examined the effect of *in vitro *priming with lithium chloride on the phenotypic differentiation of transplanted adult NPCs. In particular, we examined the effect of lithium chloride on the generation of DARPP-32 positive cells, a marker of striatal medium-sized spiny neurons representing the main neuronal cell type lost in HD. Although the proportion of BrdU-labeled cells coexpressing NeuN was not altered by lithium chloride priming of adult NPCs, we did observe a significant increase (*t*_21 _= 2.23; *P *= 0.035; Figure 3e) in the proportion of BrdU-labeled cells coexpressing DARPP-32 in rats that received lithium-primed (9.37 ± 0.98%) compared with nonprimed (6.99 ± 0.55%) adult NPCs transplants (Figure 3a, b, d).

These results indicate that *in vitro *priming of adult NPCs with lithium chloride can reduce the generation of glial progeny after transplantation into the QA-lesioned striatum. Although lithium chloride priming alone did not increase the overall proportion of mature neurons generated from transplanted adult NPCs, our results suggest that lithium chloride priming is able to enhance the phenotypic differentiation and/or maturation of regionally specific DARPP-32 neurons in the QA-lesioned striatum.

### Formation of efferent projections

To assess whether transplanted adult NPCs formed anatomically appropriate efferent projections, rats received an injection of the retrograde tracer Fluorogold (FG) into the globus pallidus 11 weeks after transplantation. BrdU/DARPP-32/FG (Figure 3a through 3d) co-labeled neuronal soma were identified in the lesioned striatum of animals that received either nonprimed or lithium chloride-primed adult NPC transplants. Quantitative analysis determined that lithium chloride-primed transplants produced significantly more BrdU/FG-colabeled projections to the globus pallidus than did nonprimed transplants by approximately 60% (nonprimed, 6.90 ± 1.14%; primed, 11.26 ± 1.52%; *t*_18 _= 2.21; *P *= 0.040; Figure 3f). FG is not taken up by fibers of passage [27,28]. Therefore, it can be proposed that BrdU/DARPP-32/FG-colabeled soma in the QA-lesioned striatum had processes that terminated in the globus pallidus. These findings further indicate that *in vitro *priming of adult NPCs with lithium chloride enhances neuronal maturation of transplanted adult NPCs *in vivo *and augments the formation of anatomically appropriate efferent projections from the damaged host striatum to the globus pallidus.

### Sensorimotor-function analysis

Rats were tested for spontaneous exploratory forelimb use and ipsilateral retrieval bias to assess whether transplantation of lithium chloride-primed adult NPCs could augment sensorimotor-function improvement compared with transplantation of nonprimed adult NPCs.

#### Spontaneous exploratory forelimb use

**Figure 4:**
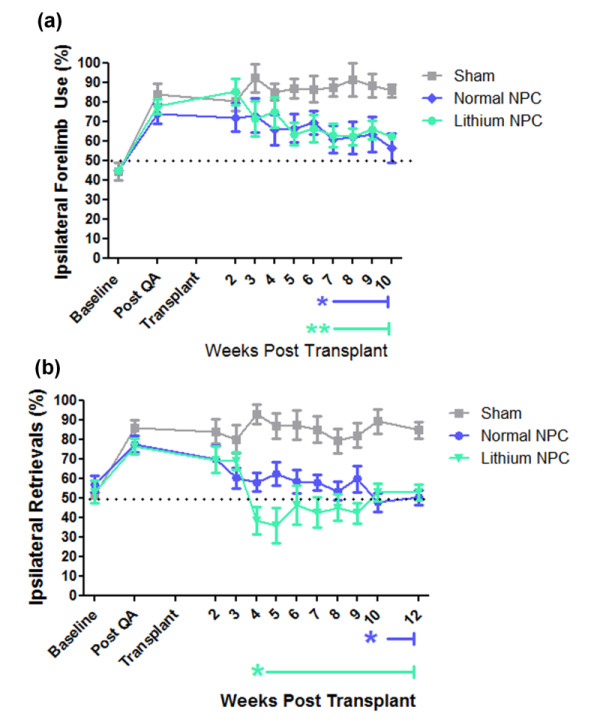
**Transplantation of lithium chloride-primed SVZ-derived adult neural progenitor cells accelerated the onset of sensorimotor-function recovery after QA striatal lesioning compared with rats that received nonprimed transplants**. Unilateral lesioning of the striatum results in lateralized deficits to the rats' sensorimotor behavior that was quantitatively assessed in **(a) **the spontaneous exploratory forelimb-use test, and **(b) **sensorimotor-neglect corridor task to monitor the development of functional impairments after QA injection and functional improvement after adult SVZ-derived neural progenitor-cell transplantation. Significance designated by repeated-measure two-way analysis of variance (ANOVA) with Bonferroni *post hoc *analysis relative to sham transplant. *P < 0.05; **P < 0.01.

After QA lesioning, all treatment groups exhibited a strong preference for use of the ipsilateral forelimb compared with baseline (*F*_(1, 24) _= 287.0; *P *< 0.0001; two-way RM ANOVA, baseline and post-QA time points only; Figure 4a). After transplantation, no change in ipsilateral forelimb use in sham (media only) transplanted animals was noted across the duration of the study (*F*_(9, 45) _= 0.68; *P *= 0.72; RM one-way ANOVA; Figure 4a). However, in animals that received a transplant of adult NPCs, two-way repeated-measure ANOVA comparing sham with either priming status at each time point confirmed a significant effect of transplantation on forelimb asymmetry (*F*_(2, 180) _= 3.62; *P *= 0.008) and an interaction between transplantation and time (*F*_(20, 180) _= 1.57; *P *= 0.01) with transplanted animals, showing decreased ipsilateral forelimb use over time (Figure 4a). In these animals, Bonferroni *post hoc *analysis identified the onset of a sustained and significant reduction in ipsilateral forelimb use in NPC-transplanted compared with sham-transplanted animals from 7 weeks after transplantation (*P *< 0.05; Figure 4a). In animals that received lithium chloride-primed transplants, a sustained and significant reduction in ipsilateral forelimb use compared with sham animals was also identified at 7 weeks after transplant (*P *< 0.01; Figure 4a). No significant difference in onset or extent of motor-function recovery was identified through Bonferroni analysis of spontaneous exploratory forelimb use between rats receiving transplants of either nonprimed or lithium chloride-primed NPC transplants (*P *> 0.05).

#### Corridor Task

QA-lesioned animals in each treatment group showed a strong ipsilateral retrieval bias/contralateral sensorimotor neglect in corridor-task performance (*F*_(1, 24) _= 207.1; *P *< 0.0001, two-way RM ANOVA, baseline and post-QA time points only; Figure 4b). As with spontaneous exploratory forelimb-use analysis, no spontaneous recovery was seen in sham-transplanted animals across the 12-week period after transplant (*F*_(9, 88) _= 0.61; *P *= 0.78; one-way RM ANOVA; Figure 4b). Rats that received either nonprimed or lithium chloride-primed adult NPC transplants showed a recovery from lesion-induced ipsilateral bias with a return to baseline scores by 12 weeks after transplantation (*F*_(1, 21) _= 0.74; *P *= 0.54 baseline versus 12 weeks after transplant; Figure 4b). Both nonprimed and lithium chloride-primed adult NPC transplants eventually resolved ipsilateral bias (effect of transplant: *F*_(2, 198) _= 16.84; *P *< 0.0001; interaction between time and transplant: *F*_(22, 198) _= 2.62; *P *< 0.0001; two-way RM ANOVA; Figure 4b). Bonferroni *post hoc *tests identified that animals with lithium chloride-primed NPC transplants showed a sustained and significant reduction in ipsilateral bias from 4 weeks after transplantation compared with sham-transplanted animals (*P *< 0.05). Furthermore, a significant preference for contralateral retrievals was observed at 5 weeks after transplant in rats receiving lithium chloride-primed NPC transplants compared with both sham-transplanted animals (*P *< 0.01) and animals receiving nonprimed adult NPC transplants (*P *< 0.05). In contrast, animals with nonprimed adult NPC transplants did not show a significant reduction in ipsilateral bias compared with sham-transplanted animals until 10 weeks after transplantation (*P *< 0.05).

Combined, these results suggest that transplantation of adult NPCs primed *in vitro *with lithium chloride can accelerate the onset of sensorimotor-function recovery compared with transplantation of nonprimed NPCs.

## Discussion

Predetermining the fate of transplanted NPCs and directing their maturation will form a cornerstone for enhancing the success of therapeutic transplantation. Our results demonstrate that *in vitro *priming of adult NPCs with lithium chloride before transplantation leads to a reduction in gliogenesis and enhanced occurrence of DARPP-32-positive neurons 12 weeks after transplantation into the QA-lesioned striatum. In addition, lithium chloride priming increased the formation of anatomically appropriate efferent projections and accelerated improvement in sensorimotor-function performance when compared with that of nonprimed adult NPC transplants.

Regional environmental cues present in the adult brain play a strong role in determining the lineage potential of adult NPCs. Previous studies have demonstrated that transplantation of adult NPCs into homotrophic or heterotrophic neurogenic regions of the normal brain results in site-specific neuronal differentiation [14,29-31], whereas transplantation into non-neurogenic sites, such as the striatum, results in the majority of transplanted cells differentiating into glia, with approximately 0.1% to 9% of transplanted NPCs forming neurons in either the normal brain, or in lesion models of Parkinson disease or stroke [10,12,13,15]. We previously demonstrated that SVZ-derived adult NPCs differentiate into GABAergic striatal neurons 8 weeks after transplantation into the QA-lesioned striatum [9]. Nonetheless, the majority of transplanted adult NPCs coexpressed the astrocytic marker GFAP. This indicates that microenvironmental changes induced by a CNS lesion in nonneurogenic brain regions are not sufficient to induce extensive neuronal differentiation from transplanted adult NPCs.

This led us to develop a novel mechanism by which to predirect the neuronal fate of adult NPCs before transplantation to enhance therapeutic efficiency [16]. We demonstrated that transient priming with lithium chloride during expansion of adult SVZ-derived NPCs in culture increased the proportion of cells expressing neuronal markers, while concurrently reducing glial progeny *in vitro*. Extending these observations, we now demonstrate that *in vitro *priming of adult SVZ-derived NPCs with lithium chloride before transplantation reduces glial differentiation of transplanted NPCs in the QA-lesioned striatum by nearly 60% compared with nonprimed transplants. Reducing glial differentiation from transplanted NPCs is important for optimizing therapeutic efficacy, as this reduces the potential for transplanted NPCs to contribute to gliosis. Preferential differentiation of transplanted cells into astrocytes may compound the neurologic consequences of glial scarring and impede the development and functional integration of transplant-derived neurons [32,33]; this is of particular concern for transplantation strategies targeted toward HD due to preexisting glial scarring manifested during the disease process [34-36].

In strong contrast with our previous *in vitro *observations [16], lithium chloride priming did not alter the proportion of transplanted adult NPCs expressing the mature neuronal marker, NeuN, when compared with nonprimed transplants. However, we did observe an increase in the proportion of transplanted adult NPCs expressing the striatal phenotypic marker DARPP-32 in rats that received lithium chloride-primed transplants compared with nonprimed transplants. These results suggest that although lithium chloride priming of adult NPCs may not be sufficient to enhance the proportion of newly generated neurons 12 weeks after transplantation, it may provide a mechanism by which to enhance phenotypic differentiation or maturation or both of new neurons in the lesioned striatum, possibly through enhanced expression of the neurogenic factor BDNF [37-41]. This is further supported by the observation that lithium chloride priming augmented the formation of anatomically appropriate efferent projections from the damaged host striatum to the globus pallidus.

In accordance with the effect of lithium chloride priming on the formation of efferent projections from transplanted adult NPCs, rats that received lithium chloride-primed transplants exhibited accelerated sensorimotor function recovery compared with those with nonprimed transplants. This was most prominent in the corridor task, in which rats receiving lithium chloride-primed NPCs exhibited an accelerated onset of sensorimotor-function improvement compared with rats with nonprimed transplants. Most surprising, a significant preference for contralateral retrievals was observed at 5 weeks after transplant in rats receiving lithium chloride-primed NPC transplants compared with both sham-transplanted animals and animals receiving nonprimed adult NPC transplants. Although the mechanism resulting in this contralateral recovery bias is unclear, it may reflect the effect of lithium chloride priming on efferent fiber growth and anatomic integration of transplanted cells with the host.

The molecular mechanisms by which lithium chloride priming directs the differentiation, maturation, survival (or a combination of these) of adult SVZ-derived NPCs remains to be specifically clarified. Previous studies have demonstrated that lithium chloride activates a number of cell-survival factors including the PI3-kinase/Akt signaling pathway, and neurotrophic/neuroprotective proteins such as BDNF, heat-shock protein, and Bcl-2 [42], as well as promoting neurogenesis through activation of the Wnt/MAPK, ERK-, and CREB-dependent signaling pathways [17,18,43,44].

Alternatively, lithium chloride has been shown to increase the active form of the Notch receptor and upregulate its target genes in SVZ-derived NPCs independent of GSK-3β or inositol signaling [45]. Lithium chloride is well known to act as a direct inhibitor of glycogen synthase kinase-3 (GSK-3), a component of the canonic Wnt signaling pathway. Wexler and colleagues [17] demonstrated that treatment of adult hippocampal NPCs with lithium chloride *in vitro *increased proliferation and enhanced neuronal differentiation through inhibition of GSK-3a/β, suggesting that much of lithium chlorides effect on neurogenesis may be attributable to activation of the canonic Wnt pathway. Supporting this hypothesis, Kim and colleagues [18] demonstrated that treatment of adult rats with lithium chloride increased the proportion of BrdU-labeled hippocampal NPCs cells expressing p-CREB. In addition, Su and colleagues [46] demonstrated that treatment of spinal cord-derived NPCs with lithium chloride increased proliferation, BDNF production and neuronal differentiation of NPCs *in vitro*. Inhibition of GSK-3 by lithium chloride in rat cortical neurons increases BDNF by activating BDNF promoter IV [43,44]. This suggests that the CREB-BDNF pathway is involved in the neurogenic effect observed both *in vitro *and *in vivo *after priming of adult NPCs with lithium chloride.

Based on these previous findings, we propose that inhibition of GSK-3 in adult SVZ-derived NPCs before transplantation may facilitate CREB activation and increase the expression and production of BDNF, thereby enhancing NPC differentiation or maturation or both. This is in agreement with our previous findings in which we demonstrated that ectopic expression of BDNF in the striatum promoted neuronal differentiation of transplanted adult SVZ-derived NPCs [15].

## Conclusions

These initial results demonstrate the potential for lithium chloride priming to augment neuronal phenotypic differentiation, maturation, and functional integration of transplanted adult NPCs in the QA-lesion rat model of HD. Priming NPCs toward a specific neuronal fate in a controlled *in vitro *environment before transplantation has the potential to enhance the efficiency of NPC transplantation therapy. Whereas the QA lesion model was used in this initial study to provide a rapid, reliable, and well-substantiated model of the selective and extensive striatal cell loss observed in HD, future studies using transgenic models of HD are necessary to assess whether the diseased host brain affects the long-term efficiency and survival of lithium chloride-primed adult NPC transplants. Future analysis regarding the molecular mechanisms by which lithium chloride directs neuronal differentiation of adult SVZ-derived NPCs may also allow the identification and development of more specific modulators of neuronal fate.

## Abbreviations

BDNF: brain-derived neurotrophic factor; HD: Huntington disease; NPC: neural progenitor cell; QA: quinolinic acid; SVZ: subventricular zone.

## Competing interests

The authors declare that they have no competing interests.

## Authors' contributions

EMV and BC designed the research and wrote and edited the manuscript. EMV performed experiments and collected the data.
